# Epidemiology of *Staphylococcus aureus* Bacteraemia at a Tertiary Children’s Hospital in Cape Town, South Africa

**DOI:** 10.1371/journal.pone.0078396

**Published:** 2013-10-22

**Authors:** Reené Naidoo, James Nuttall, Andrew Whitelaw, Brian Eley

**Affiliations:** 1 Paediatric Infectious Diseases Unit, Red Cross War Memorial Children’s Hospital and the School of Child and Adolescent Health, University of Cape Town, Cape Town, South Africa; 2 National Health Laboratory Services, University of Cape Town, Cape Town, South Africa; University Hospital Münster, Germany

## Abstract

**Background:**

*Staphylococcus aureus* is an important pathogen in paediatric patients with bloodstream infections. The epidemiology of *S. aureus* bacteraemia, however, has not been well documented in children in South Africa.

**Methods:**

A retrospective study was conducted at a children’s hospital in Cape Town, South Africa, to investigate the epidemiology of *S. aureus* bacteraemia from 2007-2011. The incidence, clinical presentation, risk factors, management and outcomes of methicillin sensitive *S. aureus* (MSSA) and methicillin resistant *S. aureus* (MRSA) bacteraemia were compared.

**Results:**

Over the five year study period, 365 episodes of *S. aureus* bacteraemia were identified. The annual incidence was 3.28 cases per 1000 hospital admissions. MRSA was responsible for 26% of *S. aureus* bacteraemia and 72% of nosocomial infections. Only six possible cases of community-acquired MRSA infections were described. MSSA bacteraemia was more likely to present as pulmonary and bone or joint infections, while bacteraemia without a source was the most common presentation with MRSA.  Infants, children with malnutrition, and residents of long-term care facilities were at highest risk for MRSA bacteraemia. The overall case fatality rate for *S. aureus* bacteraemia was 8.8% over five years, with MRSA being the only significant risk factor for mortality.

**Conclusion:**

The incidence of *S. aureus* bacteraemia and MRSA bacteraemia in children has remained stable over the past five years. MRSA is a predominantly nosocomial pathogen in children with *S. aureus* bacteraemia in Cape Town, South Africa.

## Introduction

*Staphylococcus aureus* is a major human pathogen that causes a wide variety of illnesses ranging from skin and soft tissue infections to life-threatening invasive disease. This highly adaptive organism is a significant source of bacteraemia, responsible for both community-acquired and nosocomial infections. Few South African studies have characterized the contribution of *S. aureus* to bacteraemia in children. In Cape Town, 11.6% of bacteraemia in hospitalized children were due to *S. aureus*, while in specific cohorts of HIV-positive children, 10.6%, 11% and 15.7% of bacteraemic events were caused by *S. aureus* [1-4]. 

Methicillin resistant *S. aureus* (MRSA) is one of the commonest causes of healthcare-associated and nosocomial infection amongst adults and children worldwide, and the proportion of MRSA has been increasing over the last few years [5]. In the only two paediatric studies in South Africa on *S. aureus* infections, 2% of isolates were reported as MRSA at Red Cross War Memorial Children’s Hospital in 1974, while a second study, focusing on endocarditis in 36 children with SAB in Johannesburg, noted that 31% of SAB were due to MRSA in 1995 [6,7]. More recently, an antimicrobial susceptibility study on blood culture isolates from children and adults in South African public hospitals in 2010, identified 30-60% of *S. aureus* to be MRSA [8]. Similarly, two international multi-centre studies reported MRSA to be responsible for 33% and 39% of *S. aureus* infections in South Africa [9,10]. 

Since the late 1990’s, community-acquired MRSA (CA-MRSA) infections have been reported to be rapidly emerging [11]. CA-MRSA isolates are often associated with a distinct antimicrobial sensitivity pattern and a different genetic background, although this distinction is becoming more blurred as cases of HA-MRSA caused by strains with genotypes similar to that described in CA-MRSA isolates have been reported [12]. The prevalence of CA-MRSA varies widely across the world. In South Africa, a study conducted in Cape Town identified 10 possible CA-MRSA amongst 100 MRSA isolates in adults and children while an adult study in Johannesburg reported 20% of MRSA bacteraemia to have originated from the community [13,14]. 

The aims of this study were to investigate the epidemiology of SAB at a single children’s hospital in South Africa over a five year period, and to describe the incidence, clinical presentation, microbiologic profiles, risk factors, management and outcomes of children with both methicillin-sensitive *S. aureus* (MSSA) and MRSA bacteraemia. 

## Materials and Methods

### Study population

This study was undertaken at Red Cross War Memorial Children’s Hospital (RCWMCH), a tertiary level children’s hospital in Cape Town, South Africa, which serves as a major referral centre for children up to 13 years. The hospital does not provide routine postnatal care or have a dedicated neonatal intensive care facility.

### Study design

A retrospective analysis was conducted of all paediatric patients seen at RCWMCH with *S. aureus* bloodstream infections between January 2007 and December 2011. All patients from whom *S. aureus* had been isolated from blood culture specimens over the five year period were identified using the National Health Laboratory Service (NHLS) database. Hospital clinical records were then retrospectively examined. Data were collected on demographics, clinical diagnosis, risk factors for infection, clinical and laboratory markers of infection, antibiotic susceptibility profiles, management of infection and clinical outcome. 

### Definitions

SAB was defined as the isolation of *S. aureus* from a blood culture specimen. Isolates were identified and tested for antimicrobial susceptibility at Groote Schuur Hospital NHLS microbiology laboratory, according to the Clinical and Laboratory Standards Institute guidelines appropriate for the years in question [15]. Isolates were identified using the VITEK 2 system (BioMérieux, France). Antibiotic susceptibility was determined using either standard Kirby Bauer disc diffusion, or the automated VITEK 2 system [16]. The D-test for inducible clindamycin resistance was performed on erythromycin resistant isolates, and D-test positive isolates were reported as clindamycin resistant. For MRSA isolates tested by disc diffusion, vancomycin MICs were determined using the E-test (BioMérieux, France). For isolates where susceptibility testing was performed using the Vitek 2, the vancomycin MIC as determined by the Vitek 2 was recorded. All *S. aureus* organisms cultured from blood specimens were considered to be clinically significant. However, multiple isolates of the same organism from the same patients were excluded from analysis if they were clinically deemed to be related to the incident infection. The isolation of *S. aureus* from a blood culture drawn more than 48 hours after admission to hospital, or drawn at readmission within 48 hours of discharge, was considered to be a nosocomial infection. Based on the definitions utilized by the Centers for Disease Control and Prevention, healthcare-associated infections were defined as those that occurred within 48 hours of admission to hospital, with at least one of the following healthcare-associated risk factors: hospitalization or surgery within one year of onset of infection, presence of an invasive device at the time of admission, resident of a long-term care facility, and history of MRSA colonization or infection [17]. Community-acquired MRSA infections were those identified within 48 hours of hospital admission, but with none of the aforementioned healthcare-associated risk factors. Clinical diagnoses during the SAB episode were classified according to the assessment of the attending physician in the patient records. HIV infection was determined by HIV PCR testing in children younger than 18 months, and by HIV ELISA testing in children over 18 months. HIV exposure was identified by history of maternal HIV infection, or a positive HIV antibody test in infants less than 18 months who tested HIV PCR negative. Leucocytosis was regarded as a white cell count above the normal range for age according to the NHLS reference standards. Malnutrition was defined according to WHO growth standards as weight-for-height or height-for-age z-score -2 SD below the mean WHO reference values. Severe malnutrition was defined as weight-for-height or height-for-age z score below -3SD of the mean, or by the presence of nutritional oedema [18].

### Statistical analysis

Weight-for-age z-scores (WAZ), height-for-age z-scores (HAZ) and weight-for-height z-scores (WHZ) were calculated using Epi-Info, version 3.5.3 (2011). Data were analysed using Stata Statistical Software version 11.1 (StataCorp, College Station, Texas). Non-parametric variables were evaluated with Wilcoxon rank-sum tests and Kruskal-Wallis tests, and categorical data with chi-square tests. Odds ratios, together with 95% confidence intervals, were utilized to describe measures of effect. For all analyses, p <0.05 was considered statistically significant. Logistic regression was performed to assess risk factors for MRSA infection and risk factors for mortality. Variables with p <0.1 on univariate analysis were selected for stepwise forward multivariate analysis.

### Ethical considerations

The study was conducted in accordance with the Declaration of Helsinki and was approved by the Departmental Research Committee of the Department of Child and Adolescent Health, and the Research Ethics Committee, Faculty of Health Sciences, University of Cape Town (HREC REF:029/2011). 

## Results

### Incidence of infection

Between January 2007 and December 2011, 365 episodes of *S. aureus* bacteraemia (SAB) were identified, 270 with MSSA (74%) and 95 with MRSA (26%). *S. aureus* was isolated on subsequent culture specimens, including blood, bone, tissue, pus, pleural, pericardial, peritoneal and synovial fluids in 143/365 episodes (39%). Basic demographic (age, gender), laboratory and microbiological data were accessible for all SAB episodes. Unfortunately, due to the retrospective design of the study, clinical and outcome data were not available for all cases. 

**Figure 1:**
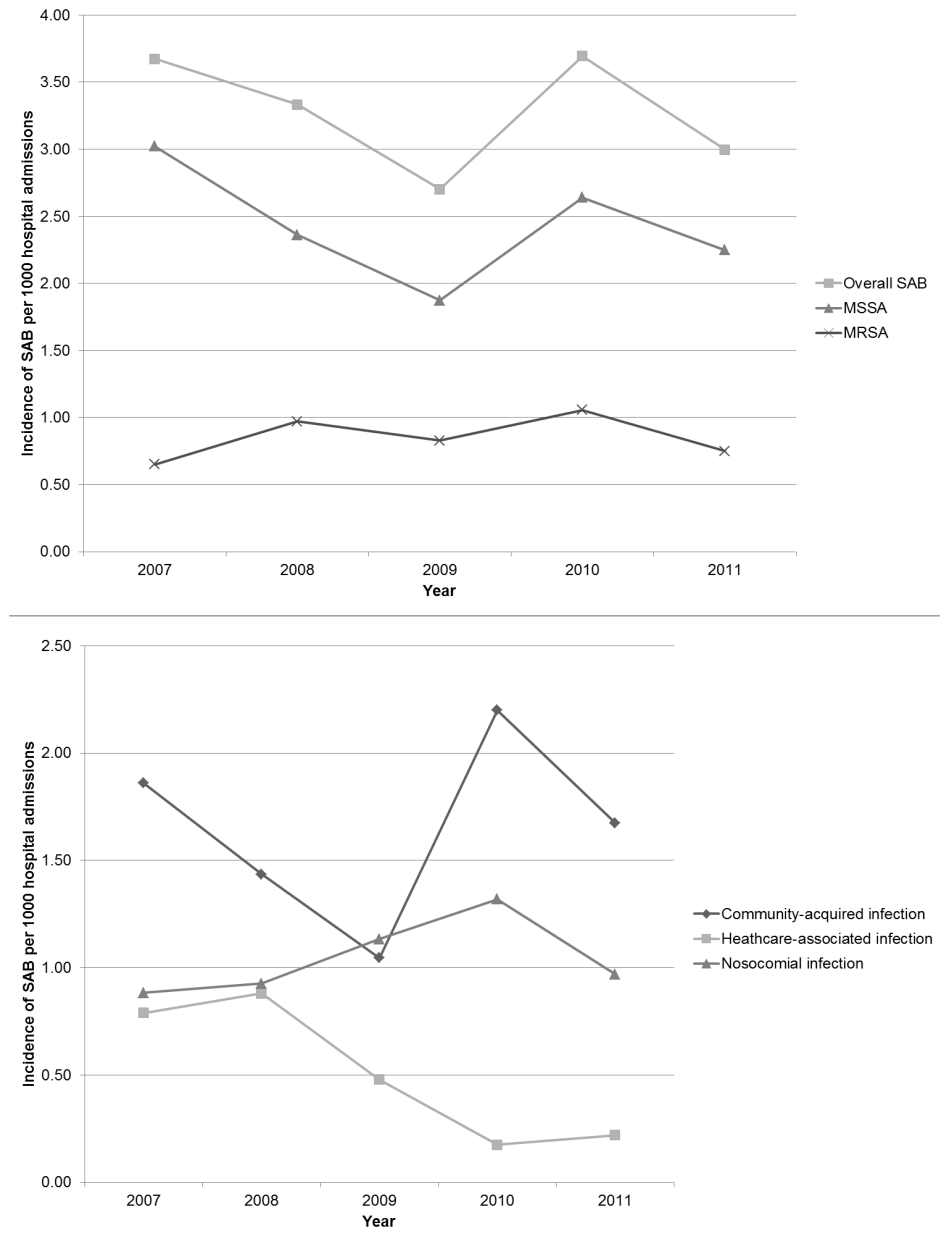
Incidence of *S. aureus* bacteraemia (SAB) per 1000 hospital admissions.

The annual incidence of SAB was 3.28 cases/1000 hospital admissions per year with a mean frequency of 2.43 MSSA cases and 0.85 MRSA cases per 1000 hospital admissions per year. There was no significant change in the frequency of SAB over the study period (p=0.38). While rates of MRSA infection remained fairly constant from 2007-2011, the incidence of nosocomial SAB rose steadily from 2007 and peaked in 2010 (Figure 1).

### Characteristics of study population

Demographic characteristics and basic infective markers of the cohort are outlined in Table 1. The median age of patients with staphylococcal bacteraemia was 11 months. Neonates accounted for 36/365 (9.9%) of episodes while infants represented 189/365 (51.8%) of SAB. Children with MRSA infection were significantly younger (p=0.03), and more frequently underweight (p=0.00) and stunted (p=0.00). In those children whose HIV exposure and testing status was known, 51 (19.8%) were HIV-infected and 48 (19.4%) were HIV-exposed but uninfected (Table 1). Notably 107 (29%) children had an unknown HIV status. HIV infection, but not HIV exposure, was significantly associated with MRSA bacteraemia (p=0.00). Analysis of infective markers, including white cell count (WCC), neutrophil count, band count and C-reactive protein (CRP), revealed no differences between MSSA and MRSA bacteraemia (Table 1). 

**Table 1:** Characteristics of patients with *S. aureus* bacteraemia.

	**All patients**	**MSSA**	**MRSA**	**p**
**Median age in months (IQR)**	(n=365)	(n=270)	(n=95)	0.03
	11.3 (3.8-42.3)	14.0 (3.7-51.1)	7.1 (3.8-19.6)	
**Male: Female**	193:172	151:119	42:53	0.05
**Median weight-for-age z-score (IQR)**	(n=331)	(n=249)	(n=82)	0.00
	-1.53 (-3.09;-0.36)	-1.24 (-2.35;-0.17)	-3.00 (-4.35;-1.38)	
**Underweight for age (%)**	(n=331)	(n=249)	(n=82)	0.00
	131 (39.6%)	79 (31.7%)	52 (63.4%)	
**Median height-for-age z-score (IQR)**	(n=167)	(n=103)	(n=64)	0.00
	-1.69 (-3.11;-0.6)	-1.39 (-2.23;-0.5)	-2.65 (-3.82;-1.01)	
**Stunted (%)**	(n=167)	(n=103)	(n=64)	0.00
	76 (45.5%)	35 (34%)	41 (64.1%)	
**Median weight-for-height z-score (IQR)**	(n=149)	(n=88)	(n=61)	ns
	-1.53 (-3.12;0.21)	-1.40 (-3.13;0.4)	-1.94 (-3.08;-0.01)	
**HIV infection (%)**	(n=258)	(n=179)	(n=79)	0.00
	51 (19.8%)	26 (14.5%)	25 (31.6%)	
**HIV exposed but uninfected (%)**	(n=248)	(n=180)	(n=68)	ns
	48 (19.4%)	39 (21.7%)	9 (13.2%)	
**Median white cell count x 109/L (IQR)**	(n=354)	(n=262)	(n=92)	ns
	14.2 (9.3-20.8)	14.3 (8.9-20.4)	13.8 (9.7-21.6)	
**Leucocytosis (%)**	(n=354)	(n=262)	(n=92)	ns
	157 (44.4%)	123 (47%)	34 (37%)	
**Median neutrophil count x 109/L (IQR)**	(n=277)	(n=204)	(n=73)	ns
	5.6 (2.9-10.6)	5.5 (2.9-10.4)	5.7 (2.5-11.2)	
**Median band count x 109/L (IQR)**	(n=268)	(n=198)	(n=70)	ns
	1.7 (0.48-3.6)	1.7 (0.47-3.27)	1.9 (0.49-4.3)	
**Median CRP mg/L (IQR)**	(n=193)	(n=146)	(n=47)	ns
	53.6 (15.2-175.5)	54.5 (14.4-176.7)	48.8 (16.1-134)	
**Median temperature at time of blood culture °C (IQR**)	(n=321)	(n=246)	(n=75)	ns
	38°C (37.0-38.8°C)	38.0°C (36.9-38.8°C)	38.1°C (37.3-38.9°C)	

(IQR = Interquartile range; CRP = C-reactive protein; ns= Not significant)

Seventeen patients had two distinct episodes of SAB based on isolation of the second organism more than 30 days after the initial SAB episode and after completing an appropriate course of antibiotic therapy. Six of these patients first had MSSA followed by MRSA bacteraemia with a median of 51 days (range 4-278 days) between episodes. The remaining 11 patients are described in Table 2. Six patients had two episodes of SAB more than 3 months apart (median 221 days; range 101-807 days). The remaining 5 patients had a median of 64 days (range 32-64 days) between the two episodes of bacteraemia. However, as molecular typing was not available, it was not possible to determine if these episodes were due to re-infection with the same type of organism or a completely new *S. aureus* infection.

**Table 2:** Characteristics of patients with two episodes of *S. aureus* bacteraemia.

**Duration between SAB episodes (days)**	**Type of SAB**	**Clinical diagnosis at time of first SAB**	**Therapy prescribed**	**Clinical diagnosis at time of second SAB**	**Underlying chronic illness**
64	MSSA	BSI no source	Cloxacillin IV 7 days	Meningitis	Hydrocephalus
205	MRSA	Pneumonia	Vancomycin IV 31 days	Pneumonia	Cystic fibrosis
807	MSSA	CVC-related infection	Cloxacillin IV 9 days; PO 19 days	BSI no source	Nephrotic syndrome
139	MSSA	BSI no source	Cloxacillin IV 15 days	BSI no source	CCD
364	MSSA	BSI no source	Ceftriaxone IV 7 days	BSI no source	Malnutrition
34	MRSA	Drip site sepsis	Vancomycin IV 21 days	BSI no source	Malnutrition; HIV
238	MSSA	BSI no source	Cefotaxime IV 5 days; Cloxacillin PO 15 days	BSI no source	Chronic renal failure; Renal transplant
34	MRSA	Sternal wound sepsis	Vancomycin IV 14 days	BSI no source	CCD
32	MRSA	BSI no source	Vancomycin IV 16 days	BSI no source	Preterm infant; Malnutrition
101	MRSA	CVC-related infection	Vancomycin IV 11 days	BSI no source	Short bowel syndrome
63	MRSA	BSI no source	Vancomycin IV 16 days	BSI no source	Measles; Tracheostomy

BSI=Bloodstream infection; CVC=Central venous catheter; CCD=Congenital cardiac disease; IV=Intravenous; PO=Per os

### Spectrum of *S. aureus* infections

The origin of SAB, was identifiable in 357/365 episodes: 33% (118/357) were nosocomial, 16% (56/357) healthcare-associated and 51% (183/357) community-acquired. MRSA was responsible for 21% of healthcare-associated and 72% of nosocomial infections. Six MRSA infections were classified as community-acquired with no identifiable healthcare-associated risk factors (one in 2007, one in 2009, three in 2010 and one in 2011). Primary diagnoses for these suspected CA-MRSA cases were bacteraemia without identified source (4), gastroenteritis (1) and superficial skin sepsis (1). 

Data on clinical diagnosis, available for 337 episodes of SAB (Table 3), showed that the most common clinical diagnosis was SAB without an identifiable source (33%), followed by SAB with pneumonia ± empyema (22%), SAB with skin and soft tissue infections (17%) and SAB with bone or joint infections (12%). Bloodstream infections without a source were more likely to be MRSA infections, while pulmonary, bone and joint infections were more likely to be due to MSSA. The only two bone or joint infections caused by MRSA were nosocomial in origin. Central venous catheter-related line sepsis was equally likely to be attributable to MSSA or MRSA, although all were nosocomial in origin (Table 3).

**Table 3:** Clinical diagnosis at the time of *S. aureus* bacteraemia.

**Clinical diagnosis at the time of culture**	**All**	**MRSA**	**MSSA**	**OR (95%CI)**
Bloodstream infection with no source	110 (32.6%)	41 (50%)	69 (27.1%)	2.70 (1.56-4.65)*
Pneumonia & Empyema	73 (21.7%)	10 (12.2%)	63 (24.7%)	0.42 (0.18-0.89)*
Skin and soft tissue infections	58 (17.2%)	18 (22%)	40 (15.7%)	1.51 (0.76-2.91)
Bone and joint infections	39 (11.6%)	2 (2.4%)	37 (14.5%)	0.15 (0.02-0.60)*
Gastroenteritis	20 (5.9%)	2 (2.4%)	18 (7.1%)	0.33 (0.04-1.43)
Staph scalded skin syndrome	11 (3.3%)	0	11 (4.3%)	-
Central venous catheter-related infection	11 (3.3%)	6 (7.3%)	5 (2%)	3.95 (0.97-16.74)
Endocarditis	8 (2.4%)	2 (2.4%)	6 (2.4%)	1.04 (0.10-5.95)
Meningitis	2 (0.6%)	1 (1.2%)	1 (0.4%)	3.14 (0.04-247.1)
Pericardial effusion	2 (0.6%)	0	2 (0.8%)	-
Other	3 (0.9%)	0	3 (1.2%)	-
TOTAL	337 (100%)	82 (100%)	255 (100%)	-

* (p<0.05)

### Risk factors for MRSA infection

Univariate analysis showed that prior hospitalization within one year, presence of an indwelling medical device, previous MRSA infection, being a resident of a long-term care facility, HIV infection, malnutrition, congenital cardiac disease, concurrent tuberculosis, duration of incident hospitalization and infancy (under 12 months), were significant risk factors for MRSA infection (Table 4). On multivariate analysis, infancy, resident of a long-term care facility, malnutrition, and longer duration of incident hospitalization remained significant risk factors. 

**Table 4:** Risk factors for methicillin-resistant *S. aureus* (MRSA) bacteraemia.

**Risk factors for MRSA infection**	**Univariate Analysis**		**Multivariate analysis**	
	**Odds Ratio (95% CI)**		**Odds Ratio (95% CI)**	
Hospitalization within 1 year	2.89	(1.79-4.68)*	0.92	(0.41-2.06)
Surgery within 1 year	1.65	(0.94-2.91)	2.28	(0.73-7.10)
Indwelling device	2.02	(1.09-3.74)*	0.87	(0.25-3.10)
Prior MRSA infection	16.8	(4.7-60.11)*	6.54	(0.91-46.98)
Resident of a care facility	12.68	(4.04-39.76)*	47.94	(7.0-326.8)*
Gender	1.6	(0.99-2.56)	1.48	(0.69-3.14)
Age (infant vs. children >1 year)	2.05	(1.26-3.34)*	2.39	(1.07-5.38)*
Duration of incident hospitalization	1.06	(1.04-1.08)*	1.06	(1.03-1.09)*
HIV-infected	2.72	(1.45-5.12)*	1.84	(0.72-4.69)
HIV-exposed but uninfected	0.55	(0.25-1.21)	-	
Malnutrition	4.45	(2.65-7.5)*	3.12	(1.40-6.96)*
Congenital cardiac disease	2.65	(1.30-5.38)*	0.37	(0.09-1.61)
Concurrent tuberculosis	6.76	(2.24-20.39)*	1.40	(0.23-8.44)
Chronic lung disease	1.53	(0.28-8.51)	-	
Chronic renal disease	0.75	(0.21-2.74)	-	
Malignancy	2.74	(0.89-8.38)	-	
Burns	1.23	(0.37-4.01)	-	
Long-term steroid/immunosuppressant use	1.54	(0.38-6.29)	-	

(*p<0.05)

### Microbiologic profile

Results of antimicrobial susceptibility testing, available for all 365 isolates (Table 5), suggested that most MSSA isolates were susceptible to the majority of antibiotics tested. Only 6% of MSSA were susceptible to penicillin. Most MRSA isolates displayed multi-drug resistance (Table 5). All MRSA were sensitive to vancomycin (minimal inhibitory concentration (MIC) ≤ 2μg/ml); three isolates were reported to have a vancomycin MIC equal to 2μg/ml. All three cases were treated successfully – catheter-related sepsis in a preterm infant with vancomycin alone, pneumonia in an HIV-infected child with vancomycin and clindamycin used sequentially, and bacteraemia without source in a child with short bowel syndrome with a combination of vancomycin and trimethoprim-sulfamethoxazole (TMP/SMX). Compared to HIV-uninfected children, organisms isolated from HIV-infected patients were more likely to be resistant to TMP/SMX (63% vs. 21%) (p=0.00), gentamicin (55% vs. 24%) (p=0.00) and rifampicin (51% vs. 16%) (p=0.00). Of the 32 HIV-infected patients with TMP/SMX resistant isolates, 22 (69%) were on TMP/SMX prophylaxis for *Pneumocystis jirovecii* at the time of SAB. Antimicrobial resistance patterns were similar in children who were HIV-exposed but uninfected, compared to children who were not exposed to HIV. 

**Table 5:** Antimicrobial susceptibility results of methicillin-sensitive *S. aureus* (MSSA) and methicillin-resistant *S. aureus* (MRSA) isolates and risk factors for mortality with *S. aureus* bacteraemia.

**Antibiotic**	**Number (%) of resistant isolates**							
	**MSSA**	**Number of isolates tested**		**MRSA**		**Number of isolates tested**		
Penicillin	255 (94.4)	270		95 (100)		95		
Cloxacillin	0 (0)	270		95 (100)		95		
Clindamycin	15 (5.6)	269		77 (81.9)		94		
Erythromycin	18 (6.7)	269		80 (85.1)		94		
TMP/SMX	29 (10.7)	269		57 (60.6)		94		
Vancomycin	0 (0)	269		0 (0)		95		
Ciprofloxacin	4 (1.5)	267		39 (41.9)		93		
Gentamicin	22 (8.1)	268		70 (75.3)		93		
Rifampicin	7 (2.6)	268		55 (59.1)		93		
Fusidic acid	0 (0)	266		3 (3.2)		94		
Moxifloxacin	0 (0)	28		4 (8.3)		48		
Linezolid	0 (0)	128		0(0)		50		
Teicoplanin	0 (0)	36		0 (0)		22		
Tigecycline	0 (0)	28		0 (0)		19		

Of the six MRSA isolates that were suspected to be community-acquired, antimicrobial susceptibility patterns showed that 4/6 (67%) were resistant to clindamycin and erythromycin, 2/6 (33%) were resistant to TMP/SMX, 3/6 (50%) were resistant to gentamicin, and 2/6 (33%) were resistant to rifampicin. All six isolates were sensitive to vancomycin, ciprofloxacin and fusidic acid.

In 84/365 (23%) of SAB episodes, a concomitant organism was isolated on blood culture. Once typical skin contaminants (coagulase negative staphylococci, *Bacillus spp.* and *Corynebacterium* spp.) were excluded, 24/365 cultures (7%) had a clinically significant second organism (polymicrobial bacteraemia) – gram negative bacilli (10), *Enterococcus faecalis or faecium* (9) and *Streptococcus pyogenes* or *agalactiae* (5).. Infants (11%), children with malnutrition (10.5%) and those with MRSA bacteraemia (12.6%) were more likely to have a significant concomitant organism on culture. No association was found between polymicrobial bacteraemia and HIV infection or HIV exposure on chi-square analysis. 

### Antibiotic management

Data on appropriate antibiotic therapy, which was regarded as the administration of antibiotics to which the organism was sensitive, were available for 322 episodes. At the time the culture was drawn, 31/322 (9.6%) were already on antibiotics to which the organism was sensitive and likely to respond clinically. The majority of these episodes (29/31; 94%) were caused by MSSA and 25/31 (81%) were community-acquired infections. Twelve of these patients received a dose of intramuscular ceftriaxone for suspicion of severe bacterial infection at a primary care clinic prior to transfer to RCWMCH, in keeping with South African Integrated Management of Childhood Illness guidelines [19]. The median time on therapy before the blood culture was drawn was 6 hours (IQR 1.5-22.7 hours). 

Excluding those who were already on antibiotics at the time of culture, appropriate antibiotic therapy was instituted in 267/322 (82.9%) episodes of SAB. Time to appropriate therapy was calculated if the precise time of blood draw and time of antibiotic administration were documented. This information was available in 141 episodes. Appropriate therapy was initiated within a median time of 2.5 hours (IQR 0.7-8.5) in MSSA and 46 hours (IQR 4.7-55.3) in MRSA infections (p=0.00). The median time to initiation of therapy was 2.5 hours for community-acquired infections, 5.8 hours for healthcare-associated infections, and 10.6 hours for nosocomial infections (p=0.00). There was no significant difference in time to therapy when comparing episodes with an identified source of bacteraemia and those without. 

The remaining 24/322 episodes (7.5%) did not receive appropriate antimicrobials. In 18/24 cases, the *S. aureus* isolated was clinically regarded to be a contaminant. The remaining six untreated patients died; in 5 cases, death occurred before blood culture results became available while in one case, the MRSA isolated was considered to be a contaminant. Two deaths occurred upon arrival at RCWMCH with pulmonary infections caused by MSSA noted on autopsy. The remaining four deaths were related to MRSA bacteraemia without a source; three of these were also malnourished. 

Total treatment duration, which was known for 261 episodes, varied according to the clinical diagnosis. The overall treatment duration was 14 days (IQR 7-29 days). Endocarditis, pulmonary and bone or joint infections were treated for a median of 44 days (IQR 39-51 days), 13 days (IQR 7-23 days) and 40 days (IQR 32-79 days) respectively. Central venous catheter-related infections were treated for 16 days (IQR 13-28 days); 8/11 episodes required line removal due to recurrent isolation of *S. aureus*. SAB without a source was treated for a median of 9 days (IQR 6-14 days). 

### Outcomes

According to clinical records, 32 deaths were attributed to SAB, resulting in a case fatality rate of 8.8% over the five year period. Median time to death was 3.5 days (IQR 1.5-6.5 days). SAB without a source accounted for 59% of deaths, while pulmonary infection was responsible for an additional 25% of mortality. There were no deaths attributed to central venous catheter-related infection. MRSA accounted for 53% of deaths. Factors contributing to mortality on univariate analysis included MRSA infection (OR 3.71; CI 1.77-7.66), HIV infection (OR 2.91; CI 1.23-6.87) and malnutrition (OR 2.45; CI 1.17-5.12) (Table 6). An identifiable source of SAB infection (OR 0.3; CI 0.14-0.62), and initiating appropriate antibiotic therapy within eight hours of blood culture, had a protective effect (OR 0.32; CI 0.13-0.77). Age, polymicrobial bacteraemia and the occurrence of multiple positive cultures for *S. aureus*, were not associated with mortality. On multivariate analysis, only MRSA remained a significant risk factor for mortality (OR 3.76; CI 1.12-12.67). 

**Table 6:** Risk factors for mortality with *S. aureus* bacteraemia.

**Risk factors for Death**		**Univariate Analysis**		**Multivariate analysis**	
		**Odds Ratio (95% CI)**		**Odds Ratio (95% CI)**	
MRSA bacteraemia		3.71	(1.77-7.76)*	3.76	(1.12-12.67)*
HIV infection		2.91	(1.23-6.87)*	3.12	(0.99-9.78)
HIV exposed but uninfected		0.31	(0.07-1.40)	-	
Malnutrition		2.45	(1.17-5.12)*	1.7	(0.49-5.81)
Age		0.84	(0.40-1.74)	
Gender		1.98	(0.94-4.19)	1.6	(0.52-4.92)
Appropriate antibiotics within 8 hours of blood culture		0.32	(0.13-0.77)*	0.54	(0.17-1.67)
Identified source of infection		0.3	(0.14-0.62)*	0.39	(0.12-1.22)
Multiple positive cultures for *S. aureus*		1.22	(0.59-2.55)	-	
Polymicrobial bacteraemia		2.28	(0.81-6.44)	-	

^*^ (p<0.05)

## Discussion

This report is the first comprehensive study of the epidemiology of *S. aureus* bacteraemia in children in South Africa. International trends show increasing reports of MRSA bacteraemia in children over the last 20 years. In England and Wales, a large multicenter study, reported an increase in the proportion of MRSA bacteraemia from 0.9% in 1990 to 13% in 2000 [20]. In the United States, Wisplinghoff et al. (2003) reported MRSA bacteraemia to have increased from 10% in 1995 to 29% in 2001 in 49 paediatric hospitals throughout the country, while Burke et al. (2009) reported a similar increase of 9% in 2001 to 24% in 2006 in California [21,22]. No published data are available for Africa on the trends of SAB in children. In this study, the proportion of SAB and MRSA bacteraemia remained fairly constant over the period 2007 to 2011. However, the proportion of MRSA bacteraemia in children in South Africa has shown a distinct upward trend from 2% in 1974, to 18% in 1992, to 26% in this study [1,6]. However, this is lower than the 30-60% MRSA bacteraemia reported among adults and children in public sector hospitals in South Africa in 2010 [8]. 

Nosocomial and healthcare-associated infection accounted for approximately half of all cases of SAB. The number of cases of *S. aureus* bacteraemia acquired as nosocomial infection, increased between 2009 and 2010 possibly due to a concurrent measles epidemic in 2009-2010, and then declined in 2011. There was a marked decline in the number of healthcare-associated infections from 2008 to 2011. There was no clear reason for this decline. Changes in *S. aureus* colonization rates within the hospital may be responsible for this observation, but further investigation is required. In this study, MRSA was a predominantly nosocomial or healthcare-associated pathogen. The proportion of CA-MRSA isolates identified (6.3%), is lower than those of other South African reports [13,14]. This study, however, focused on bloodstream infections, while most cases of CA-MRSA have been reported with skin and soft tissue infections. Due to the retrospective study design, molecular typing was not available for the CA-MRSA isolates.

 As reported in other studies, infants are at greatest risk of SAB, especially with regards to MRSA bacteraemia [20,23]. In our cohort, they were also more likely to present with a concomitant organism on blood culture. This susceptibility to systemic bacterial illness may be attributed to the relative immaturity of immune responses in infants. HIV-infected children also have an increased risk of bacteraemia [24,25]. A sizable proportion of our cohort (20%) was HIV-infected, of which 49% had MRSA bacteraemia. Other South African studies also demonstrated high proportions of MRSA in HIV-infected children ranging from 77-100% [2-4]. This may be due to the fact that HIV-infected children are more likely to be hospitalized than other children. Several common infective markers were also analyzed but were unable to distinguish MSSA from MRSA bacteraemia (Table 1).

Bacteraemia without a source was the most common clinical diagnosis. All community-acquired bone or joint infections were MSSA in origin, which supports the empiric antibiotic choice of cloxacillin for osteomyelitis and septic arthritis at RCWMCH. Endocarditis was only diagnosed in 2.4% of episodes of SAB. Although children in this study were not screened with echocardiography, other studies have also shown that endocarditis with SAB is an uncommon event in children compared to adults [22,23,26]. 

As most episodes of MRSA bacteraemia were nosocomial or healthcare-associated infections, the antimicrobial sensitivity pattern reflected predominantly multi-drug resistant MRSA isolates, which is in agreement with antimicrobial susceptibility studies on both adults and children in South Africa [27,28]. All isolates were susceptible to vancomycin, which remains the drug of choice for managing MRSA infections in children. Low rates of TMP/SMX and clindamycin susceptibility, limits the use of these antibiotics until antimicrobial susceptibility results are available for individual MRSA isolates. Notably, HIV-infected children were observed to be infected with *S. aureus* isolates that were more likely to be resistant to TMP/SMX, gentamicin and rifampicin. This may be due to the widespread use of TMP/SMX in most HIV-infected children for prophylaxis against *Pneumocystis jiroveci* infection, while gentamicin is a common first line antibiotic agent for all children presenting to hospital with sepsis. Many children with HIV are treated with rifampicin for TB during their clinical course, which may account for the high rates of rifampicin resistance in this cohort. 

The median time to appropriate therapy in this study was significantly delayed for MRSA infections, but this did not impact on mortality. Identifying children with risk factors for MRSA infection allows better guidance for empiric antibiotic therapy for nosocomial and healthcare-associated infections. In this setting, infants, malnourished children, and residents at long-term care facilities should be treated with vancomycin as part of their empiric antibiotic coverage for nosocomial infection if *S. aureus* is considered likely, until results of antimicrobial sensitivities are available. The recent clinical practice guidelines, published by the Infectious Diseases Society for America for the treatment of MRSA infections in adults and children, advise treating uncomplicated SAB with at least 14 days of appropriate antibiotic therapy [29]. The treatment duration for this category of patient was observed to be shorter in our cohort. In our study it was not possible to determine if shorter courses of treatment for SAB without a source were as effective as two weeks of therapy. 

The retrospective nature of the study imposed some limitations. The number of SAB episodes reported may be an underestimate of the true incidence of disease due to non-standardized indications for blood culture, variability in blood culture volumes collected in paediatric practice, and the initiation of antibiotics in some cases prior to blood culture collection. The absence of molecular typing of MRSA isolates limited complete evaluation of the staphylococcal bacteramic events, especially description of the CA-MRSA isolates. Moreover, our study had a high rate of concomitant organisms which were not excluded from analysis, as their presence on blood culture did not preclude the presence of a significant infection with *S. aureus*.

This study presents important clinical and epidemiological information not previously available on *S. aureus* bacteraemia in children in South Africa. Although rates of SAB and MRSA bacteraemia were stable in our population, MRSA was a significant source of nosocomial infection and was a major risk factor for mortality in children with SAB. Prospective studies that combine clinical and molecular epidemiology of *S. aureus* infections in children are warranted.
